# Proteasome inhibition attenuates rasfonin-induced autophagy concurring with the upregulation of caspase-dependent apoptosis

**DOI:** 10.1080/21501203.2016.1147091

**Published:** 2016-02-26

**Authors:** Siyuan Yan, Yongsheng Che, Xuejun Jiang

**Affiliations:** a State Key Laboratory of Mycology, Institute of Microbiology, Chinese Academy of Sciences, Beijing100101, China; b State Key Laboratory of Toxicology & Medical Countermeasures, Beijing Institute of Pharmacology & Toxicology, AMMS, Beijing100850, China; c University of Chinese Academy of Sciences, Beijing100039, China

**Keywords:** Rasfonin, apoptosis, autophagy, MG132, UPS

## Abstract

Two major protein quality control mechanisms exist in eukaryotic cells, the ubiquitin-proteasome system (UPS) and the autophagy–lysosome system. Generally, the inhibition of UPS is believed to enhance autophagic pathway; nevertheless, the crosstalk between these two degradation systems may be much more complicated. Rasfonin, a 2-pyrone derivative of fungal secondary metabolites, is demonstrated to have the antitumor effect and can function as an autophagy inducer. Here, we reported that rasfonin activated multiple cell death pathways, including caspase-dependent apoptosis. Using electroscopy and microscopy, we observed rasfonin increased the formation of autophagosome. In immunoblotting assay, rasfonin enhanced autophagic flux concomitant with the upregulation of ubiquitination. MG132, an inhibitor of proteasome, attenuated rasfonin-dependent autophagy, whereas its presentation stimulated rasfonin-induced cleavage of poly (ADP-ribose) polymerase, a marker of caspase-dependent apoptosis. Together, we demonstrated that rasfonin induced the activation of both UPS and autophagic pathway, and the inhibition of UPS attenuated rasfonin-induced autophagy and enhanced the cytotoxicity of rasonin by upregulation of caspase-dependent apoptosis.

## Introduction

Rasfonin, a natural product isolated from the fermented mycelium of *Talaromyces* sp. 3656-A1, is a derivative of a class of fungal secondary metabolites known as 2-pyrones (Tomikawa et al. 2000). 2-Pyrones have been previously reported to have a broad range of biological activities including antimicrobial, antiviral, antitumor and anti-inflammatory effects (Hilgeroth 2002; Marrison et al. 2002; McGlacken & Fairlamb 2005). Coumarins, important sort of 2-pyrones derivative, target a number of pathways in cancer such as kinase inhibition, cell cycle arrest, angiogenesis inhibition and antimitotic activity (Thakur et al. 2015). Recently, rasfonin was shown to induce the death of ras-mutated pancreatic tumour (Panc-1) cells (Xiao et al. 2014).

In eukaryotic cells, there exist two major protein quality control mechanisms, the ubiquitin-proteasome system (UPS) and the autophagy–lysosome system. The UPS is a major cellular mechanism that regulates intracellular protein levels and eliminates damaged, misfolded and mutant proteins (Glickman & Ciechanover 2002). UPS substrates are covalently conjugated with ubiquitin (Ub), a highly conserved 76-residue protein, and polyubiquitinated proteins are mostly targeted to 26S proteasomes for proteolysis into small peptides of 3–24 amino acids (Pickart & Cohen 2004). MG132 is a potent, membrane-permeable proteasome inhibitor, which can inhibit proteasome activity and block the degradation of polyubiquitinated proteins (Lee & Goldberg 1998).

Macroautophagy (hereafter called as autophagy) is an evolutionarily conserved intracellular membrane trafficking process that is involved in the delivery of cytoplasmic contents and organelles to the lysosomes for degradation (Yang & Klionsky 2010). While the UPS is responsible for short-lived proteins, autophagy clears many long-lived proteins and organelles (Lee et al. 2013). A recent study shows that the 26S proteasome is degraded by autophagy in *Arabidopsis* (Marshall, Li, et al. 2015). LC3 (a mammalian homolog of yeast Atg8) is the most widely monitored autophagy-related protein. It is initially synthesized in an unprocessed form, proLC3, which is cleaved after the C-terminal glycine to form cytosolic LC3-I, followed by its subsequent conjugation to PE to generate the membrane-associated LC3-II form (Kabeya et al. 2004). Finally, LC3-II degrades via the lysosomal turnover. LC3-II is the only protein marker that is reliably associated with completed autophagosomes (Klionsky et al. 2012). Indeed, in some cases, autophagosome accumulation detected by electron microscopy does not correlate well with the amount of LC3-II. This is particularly evident in those cells that show low levels of LC3-II (based on western blotting) because of an intense autophagy flux that consumes this protein (Castino et al. 2008). So it is highly recommended to monitor dynamic autophagic flux other than steady-state methods (Klionsky et al. 2012). Chloroquine (CQ), the lysosomotropic compounds that neutralize the lysosomal pH, blocks the lysosomal turnover. Thus, CQ is widely used as an inhibitor of autophagic flux, and blocks the degradation function of autophagy (Kovacs & Seglen 1982; Grumati et al. 2010).

In the present study, we demonstrated that MG132 attenuate rasfonin-induced autophagy, accompanied with enhancing rasfonin-activated caspase-dependent apoptosis.

## Results

### Rasfonin induces multiple cell death pathways

**Figure 1.**
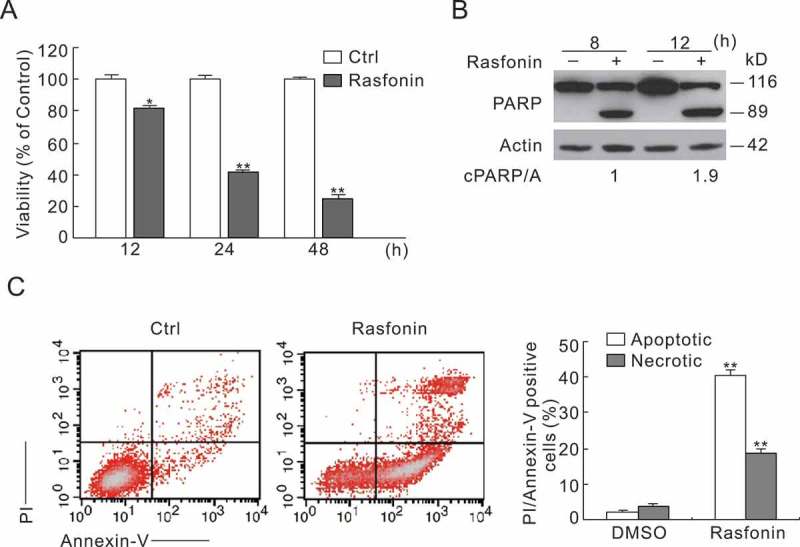
Rasfonin induces multiple cell death pathways. (A) ACHN cells were treated with rasfonin (6 μM) for up to 48 h; cell viability was analysed by MTS assay as described in ‘Materials and methods’. (B) The cells were treated with rasfonin (6 μM) upon to 12 h, and then cell lysates were prepared and analysed by immunoblotting using the indicated antibodies; actin was used as loading control. (C) Following treatment of the cells with rasfonin (6 μM) for 12 h, the apoptosis and necrosis induced were determined by flow cytometry. Apoptotic: AV-positive and PI-negative; necrotic: PI-positive. For histogram results, the data are presented as mean ± SD from three independent experiments. The single asterisk denotes the group is statistically different from the control groups (*p* < 0.05), whereas double asterisk means *p* < 0.01.

Using MTS assay, we detected the cell viability of ACHN cells following the rasfonin treatment. We found that rasfonin significantly reduced the cell viability of ACHN cells in a time-dependent manner (Figure 1A). poly (ADP-ribose) polymerase (PARP-1) is one of the main cleavage targets of caspase-3 *invivo*, and cleavage of PARP-1 serves as a marker of cells undergoing apoptosis (Amé et al. 2004; Andrabi et al. 2006). At both 8 and 12 h time points, we observed that rasonin induced the cleavage of PARP-1 (Figure 1B), suggesting that rasfonin activates apoptotic pathway. Using flow cytometry, we found that rasfonin-induced cell death could be either apoptotic or necrotic (Figure 1C) (Zhang et al. 2011; Klionsky 2012).

### Rasfonin stimulates autophagy in ACHN cells

**Figure 2.**
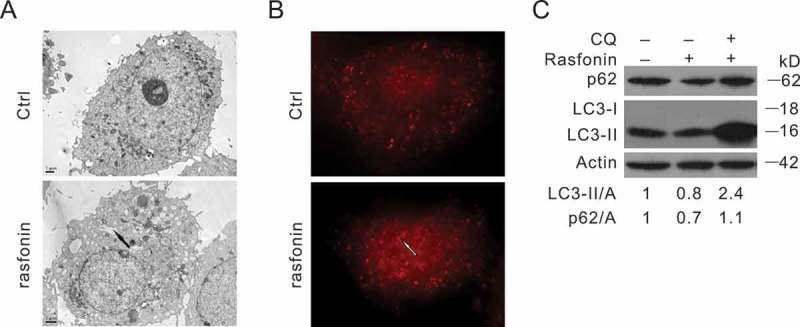
Rasfonin stimulates autophagy in ACHN cells. (A) Electron microscopy was performed ACHN cells following treatment of rasfonin (6 μM) for 2 h. (B) Immunofluorescence using LC3 antibody was performed ACHN cells following treatment of rasfonin (6 μM) in the presence or absence of CQ (10 μM) for 2 h. The area indicated by the arrow represents the autophagosome. (C) Following treatment of rasfonin (6 μM) for 2 h in the presence or absence of CQ (10 μM), ACHN cells were lysed and subjected to immunoblotting with the antibodies indicated. Densitometry was performed for quantification and the adjusted ratio of LC3-II and p62 to actin (A) were presented below the blots. Data represent three independent experiments.

Electron microscopy, one of the most valid and important method to qualitatively and quantitatively monitor various autophagic structures, was performed in rasfonin-treated ACHN cells (Klionsky et al. 2012). Compared with the dimethyl sulphoxide control, rasfonin caused an obvious accumulation of membrane vacuoles (Figure 2A). The endogenous LC3 was labelled *in situ* by immunofluorescence and then observed with fluorescence microscopy. Compared with the control cells, rasfonin markedly increased the punctate staining of LC3 (Figure 2B), indicating that rasfonin induced the formation of autophagosomes. To detect autophagic flux, the level of LC3-II was measured in the presence of CQ by immunoblotting. Although rasfonin decreased LC3-II levels at the 2 h time point, CQ addition increased the accumulation of LC3-II (Figure 2C). Additionally, rasfonin reduced the expression of p62/SQSTM1, a selective substrate of autophagy (Klionsky et al. 2012), and CQ blocked the rasfonin-induced p62 degradation, suggesting an enhanced autophagic flux (Figure 2C). The aforementioned results indicated that rasfonin was able to stimulate autophagic process in this cell line.

### MG132 diminishes rasfonin-dependent autophagy

**Figure 3.**
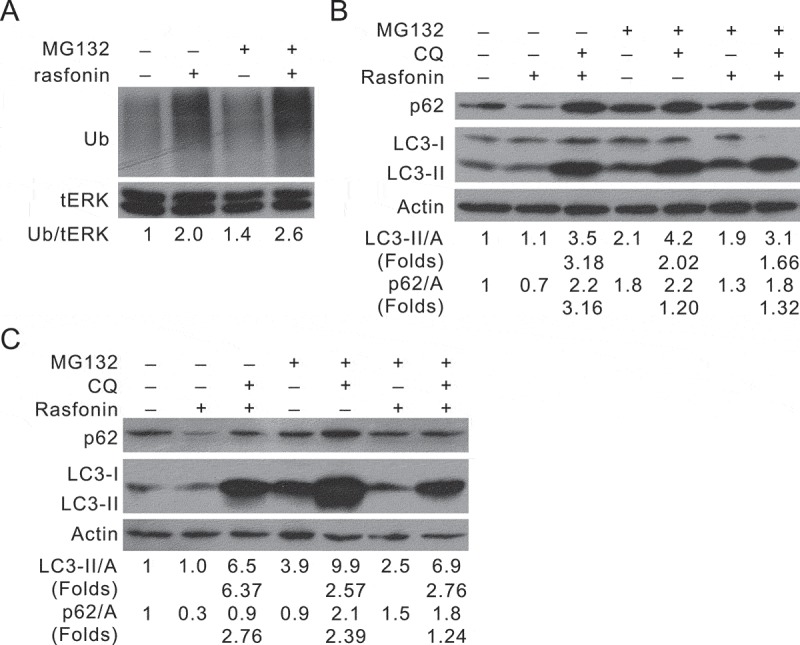
Proteasome inhibitor MG132 diminishes rasfonin-stimulated autophagy. ACHN cells were treated with rasfonin (6 μM), or MG132 (0.5 μM), or the combination of rasfonin (6 μM) and MG132 (0.5 μM) in the presence or absence of CQ (10 μM) upon to 12 h (A and B: 2 h; C: 12 h). Cell lysates were prepared and analysed by immunoblotting using the indicated antibodies. The adjusted ratios of LC3-II and p62 to actin (A), Ub to tERK1/2 were presented below the blots. Data represent three independent experiments.

Two major protein quality control mechanisms exist in eukaryotic cells, UPS and the autophagy–lysosome system, and there is a crosstalk between them. Thus, we next examine whether rasfonin can activate USP. As shown in Figure 3A, rasfonin increased ubiquitination, suggesting that rasfonin can activate both USP and autophagic pathway. Ub is a conserved polypeptide unit that plays an important role in the Ub–proteasome pathway. As an important unit in the Ub–proteasome pathway, Ub covalently linked many cellular proteins by the ubiquitination process, which targets proteins for degradation by the 26S proteasome (Ciechanover 1998). MG132, a widely used proteasome inhibitor, was reported to stimulate proteaphagy, a process that describes autophagy affects the UPS by eliminating 26S proteasomes (Marshall, Li, et al. 2015). Consistently, we observed that MG132 induced autophagy in ACHN cells, as CQ further increased the ratio of LC3-II to actin, and blocked the p62 degradation (Figure 3B). Interestingly, MG132 reduced rasfonin-induced autophagy, as judging LC3-II accumulation and p62 degradation ratios in the presence of CQ (Line 7/Line 6 versus Line 3/Line 2) at both 2 and 12 h time points (Figure 3B and 3C).

### MG132 enhances rasfonin-induced the cleavage of PARP-1

**Figure 4.**
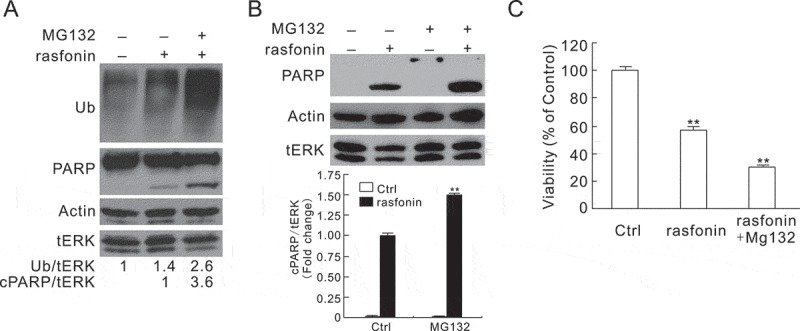
MG132 enhances rasfonin-induced PARP-1 cleavage. (A and B) Following treatment of the cells with rasfonin (6 μM) upon to 12 h (A: 8 h; B: 12 h) in the presence or absence of MG132 (0.5 μM), immunoblotting was carried out with the indicated antibodies; tERK1/2 was used as loading control. Densitometry was performed for quantification and relative ratios of Ub and cleaved PARP (cPARP) were shown below the blots. (C) ACHN cells were treated with rasfonin (6 μM) for 24 h in the presence or absence of MG132 (0.5 μM), cell viability was analysed by MTS assay. Double asterisk means *p* < 0.01. Similar experiments repeated three times.

Former studies indicated that MG132 also involved in apoptosis, playing an either promotion or inhibition role (Grimm et al. 1996; Nencioni et al. 2006). Therefore, we detected the influence of MG132 on rasfonin-induced apoptosis in the following experiments. In immunoblotting assay, we observed that MG132 blocked rasfonin-induced polyubiquitinated proteins degradation and increased rasfonin-induced PARP-1 cleavage by 3.6-fold at the 8 h time point (Figure 4A), suggesting an enhanced apoptotic process. In contrast, MG132 alone did not induce the cleavage of PARP-1 even at the 12 h time point (Figure 4B). However, MG132 was found to promote rasfonin-dependent PARP-1 cleavage at this time point (Figure 4B). In addition, compared with rasfonin alone, the combination of MG132 with rasfonin showed synergistic inhibition on cell viability at the 24 h time point (Figure 4C). All the aforementioned results indicate that MG132 enhanced the cytotoxicity of rasfonin.

## Materials and methods

### Chemicals and antibodies

Chloroquine diphosphate salt (CQ, C6628), MG132 (M8699) and polyclonal antibodies against LC3 (L7543) were purchased form Sigma-Aldrich (St. Louis, MO, USA). Antibodies against PARP (9542) and p44/42 MAPK (total-Erk1/2, 9102) were purchased from Cell Signaling Technology (Beverly, MA, USA). Antibodies of Ub (sc-8017) and p62 (sc-28359) were acquired from Santa Cruz Biotechnology (Santa Cruz, CA, USA). Antibody against actin (TA-09) was obtained from ZhongShanJinQiao Biocompany (Beijing, China). MTS reagent powder (G1111) was acquired from Promega Corporation (Madison, WI, USA).

### Cell culture and immunoblotting analysis

ACHN cell lines were grown in DMEM medium containing 10% fetal bovine serum (GIBCO), and 1% antibiotics. Cells were grown to 70% confluence before adding varieties of compounds. Whole cell lysates were prepared with lysis using Triton X-100/glycerol buffer, containing 50 mM Tris-HCl, pH 7.4, 4 mM EDTA, 2 mM EGTA and 1 mM dithiothreitol, supplemented with 1% Triton X-100, 1% SDS and protease inhibitors and then separated on a SDS-PAGE gel and transferred to PVDF membrane. Immunoblotting was performed using appropriate primary antibodies and horseradish peroxidase-conjugated suitable secondary antibodies, followed by detection with enhanced chemiluminescence (Pierce Chemical, Rockford, IL, USA).

### Cell viability assay (MTS)

Cells were plated in 96-well plates (5000 cells per well) in 100 µl complete culture medium. After overnight culture, the medium was replaced with Phenol-Red-free complete medium that was either drug-free or contained rasfonin or other chemicals. The cells were cultured for various periods and cellular viability was determined with CellTiter 96 Aqueous Non-Radioactive Cell Proliferation Assay (Promega).

### Flow cytometry assay

ACHN cells were treated with the indicated compounds, then trypsinized and harvested, washed with phosphate buffer saline (PBS) buffer, followed by incubating with a fluorescein isothiocyanate-labelled annexin V (FITC) and propidium iodide according to the instructions of an Annexin-V-FITC Apoptosis Detection Kit (Biovision Inc., Milpitas, CA, USA, K101-100) and analysed by flow cytometry (FACSAria, Becton Dickinson, Franklin Lakes, NJ, USA).

### Immunofluorescence

ACHN cells were plated on glass coverslips and then performed indicated treatment. Then cells were washed with Ca^2+^-free and Mg^2+^-free PBS (CMF-PBS), fixed with freshly prepared 4% paraformaldehyde and permeabilized incubation with CMF-PBS containing 0.1% TritoX-100 and 0.5% bovine serum albumin (BSA). Then they were washed three times with CMF-PBS, and incubated with indicated antibodies in the presence of 0.1% TritoX-100 and 0.5% BSA. After washed for three times, cells were incubated with the secondary antibodies (Alexa Fluor® 594 Goat anti-Rabbit and Alexa Fluor® 488 Goat anti-Mouse) diluting in CMF-PBS containing 0.5% BSA. The cells were then immersed in VECTASHIELD with 4',6-diamidino-2-phenylindole (H1200, Burlingame, CA, USA) to visualize the nuclei after washing for three times. Images were acquired via fluorescence microscopy.

### Electron microscopy

Electron microscopy was performed as described. Briefly, samples were washed three times with PBS, trypsinized and collected by centrifuging. The cell pellets were fixed with 4% paraformaldehyde overnight at 4°C, post-fixed with 1% OsO4 in cacodylate buffer for 1 h at room temperature, and dehydrated stepwise with ethanol. The dehydrated pellets were rinsed with propylene oxide for 30 min at RT and then embedded in Spurr resin for sectioning. Images of thin sections were observed under a transmission electron microscope (JEM1230, Japan).

### Statistical analysis

Several X-ray films were analysed to verify the linear range of the chemiluminescence signals and the quantifications were carried out using densitometry. Normally distributed data are shown as mean ± SD and were analysed using one-way analysis of variance and the Student-Newman–Keuls post-hoc test. Data are shown as mean ± SD in graphs.

## Discussion

A new finding in the present study is that proteasome inhibition probably plays a dual role in the regulation of autophagy. While MG132 alone induced autophagic process, its presence attenuated rasfonin-induced autophagy. In contrast, MG132 enhanced both rasfonin-dependent apoptosis and cytotoxicity. Consistent with the previously studies, rasfonin, a fungal secondary metabolite belonging to 2-pyrones, stimulates multiple cell death pathways.

Two major protein quality control mechanisms exist in eukaryotic cells, the UPS and the autophagy–lysosome system. Recently, a study reported that there was a crosstalk between these proteolytic systems (Park & Cuervo 2013). Usually, the inhibition of one system is likely to activate another (Park & Cuervo 2013). Consistently, we also observed that MG132 alone stimulated autophagy. Interestingly, although rasfonin, or MG132 alone promoted autophagic flux, the combination of rasfonin with MG132 showed a partly inhibitory effect in the regulating of autophagy. Thus, it is likely that proteasome blockage plays a dual role in the regulation of autophagy. We hypothesise that inhibition of proteasomes by MG132-stimulated proteaphagy, selective autophagy of inactive proteasomes (Marshall & Vierstra 2015), and this selective autophagy process may show a competitive inhibition effect on the rasfonin-induced autophagy process, as rasfonin possess more robustly capacity to promote autophagy than MG132. Besides promoted autophagy, rasfonin was found to increase the level of polyubiquitinated proteins. Considering that proteasome inhibitor MG132 further increased polyubiquitinated proteins ratio, rasfonin may function as an inducer in the process of ubiquitination. Thus, similar to another autophagy inducer rapamycin (Harston et al. 2011), rasfonin can activate both UPS and autophagic pathway in ACHN cells. However, the underlying mechanism needs to be further revealed, and our next part of work will focus on it.

Previous study indicates that MG132 inhibits the PARP-1 cleavage and apoptosis in thymocyte (Grimm et al. 1996). However, another proteasome inhibitor lactacystin induced apoptosis and the cleavage of PARP-1, while pre-treatment with autophagy enhancer rapamycin attenuated lactacystin-induced apoptosis and reduced lactacystin-induced ubiquitinated protein aggregation in differentiated PC12 cells (Pan et al. 2008). Furthermore, MG132 was found to induce apoptosis in human monocyte-derived dendritic cells (Nencioni et al. 2006). Here, we clearly demonstrated that MG132-promoted rasfonin induced the cleavage of PARP-1 and functioned synergistically with rasfonin to inhibit cell viability. Therefore, we assumed that the inhibition of proteasome regulated apoptosis in a stimulus- and cell type-dependent manner.

One study reported that autophagy may play a potential prosurvival and protumorigenic role during cancer chemotherapy (Levine & Kroemer 2008). Our new finding is that MG132 partly blocked rasfonin-induced autophagy concurring with an enhanced apoptosis and cytotoxicity. These observations may shine light on the employment of fungal secondary metabolites in the therapy of cancer, and may play a role in the screening of potent antitumor compounds in the fungal secondary metabolites.
